# Carboxymethyl cellulose/sulfur-functionalized Ti-based MOF composite: synthesis, characterization, antimicrobial, antiviral and anticancer potentiality

**DOI:** 10.1186/s11671-023-03852-2

**Published:** 2023-05-22

**Authors:** Reda M. Abdelhameed, Mohamed S. Hasanin, Amr H. Hashem

**Affiliations:** 1 Applied Organic Chemistry Department, Chemical Industries Research Institute, National Research Centre, Scopus Affiliation ID 60014618, 33 EL Buhouth St., Dokki, 12622 Giza, Egypt; 2 Cellulose and Paper Department, Chemical Industries Research Institute, National Research Centre, 12622 Dokki, Cairo, Egypt; 3 Botany and Microbiology Department, Faculty of Science, Al-Azhar University, Cairo, 11884 Egypt

## Abstract

Microbial resistance is the first morbidity and mortality cause for patients as usually a secondary infection. Additionally, the MOF is a promising material that shows a nice activity in this field. However, these materials need a good formulation to enhance biocompatibility and sustainability. Cellulose and its derivatives are well as filers for this gap. In this presented work, a novel green active system based on carboxymethyl cellulose and Ti-MOF (MIL-125-NH_2_@CMC) modified with thiophene (Thio@MIL-125-NH_2_@CMC) was prepared by a post-synthetic modification (PSM) route based. FTIR, SEM and PXRD were utilized to characterize nanocomposites. In addition, transmission electron microscopy (TEM) was used to corroborate the nanocomposites' particle size and diffraction pattern as well as the DLS affirmed the size as 50 and 35 nm for MIL-125-NH_2_@CMC and Thio@MIL-125-NH_2_@CMC, respectively. The formulation of the nanocomposites was validated by physicochemical characterization techniques, while morphological analysis confirmed the nanoform of the prepared composites. The antimicrobial, antiviral and antitumor properties of MIL-125-NH_2_@CMC and Thio@MIL-125-NH_2_@CMC were assessed. Antimicrobial testing revealed that Thio@MIL-125-NH_2_@CMC possesses greater antimicrobial activity than MIL-125-NH_2_@CMC. Additionally, Thio@MIL-125-NH_2_@CMC demonstrated promising antifungal activity against *C. albicans* and *A. niger* where MICs were 31.25 and 0.97 µg/mL, respectively. Also, Thio@MIL-125-NH_2_@CMC exhibited antibacterial activity against *E. coli* and *S. aureus* where MICs were 1000 and 250 µg/mL, respectively. In addition, the results demonstrated that Thio@MIL-125-NH_2_@CMC displayed promising antiviral activity against both HSV1 and COX B4, with antiviral activities of 68.89% and 39.60%, respectively. Furthermore, Thio@MIL-125-NH_2_@CMC exhibited potential anticancer activity against MCF7 and PC3 cancerous cell lines, where IC_50_ was 93.16 and 88.45%, respectively. In conclusion, carboxymethyl cellulose/sulfur-functionalized Ti-based MOF composite was successfully synthesized which had antimicrobial, antiviral and anticancer activities.

## Introduction

Antimicrobial resistance (AMR) is considered one of the most significant problems affecting human life in last two decades [1, 2]. Resistance to antimicrobial drugs reduces the efficacy of drugs, resulting in increased morbidity and mortality [3–6]. In addition, the adverse effects of treatment protocols may affect more than the disease being treated [7, 8]. In addition, despite the development and advancement of medical therapies, the cancer cure still faces significant obstacles. Traditional cancer treatments consist of chemotherapy, radiotherapy and surgery. Early tumors respond well to radiotherapy and surgery, whereas advanced tumors require chemotherapy, which does not guarantee efficacy and may even be detrimental to normal cells. In many instances, the discovery of new drugs is reliant on drugs with elevated side effects [9–11]. So, the progress of new generation of antimicrobial, anticancer as well as antiviral agent needs is increased day by day, particularly after appearance COV-19 pandemic [12, 13].

MIL-125-NH_2_ is a common photocatalyst that is now being employed as a bioactive substance for bacterial cells. MIL-125-NH_2_ was added to g-C_3_N_4_ then coated with quaternary ammonium compound (QAC) to form positive charge surface, and this material can enhance the electrostatic attractions performance between bacterial cells and the surface of the material. The photocatalytic activity of prepared QAC@MIL-125-NH_2_@g-C_3_H_4_ toward bacteria was increased because of the cooperative effects of bacterial cell adhesion and ROS generation. Interestingly, the QAC@MIL-125-NH_2_@g-C_3_H_4_ photocatalyst gives 3.20 times of inactivation performance for Staphylococcus epidermidis within 60 min under visible light contact [14].

MIL-125-NH_2_ can chemically connect to Rh complex via modification of organic linker backbone of MOFs. The obtained modification facilitated the photo-induced electron transfer for the NADH regeneration with the yield of 66.4% in 60 min for 5 cycles [15].

MIL-125-NH_2_ was integrated into bacteria cellulose@chitosan, and the obtained composite was used as air filters. The advantage of produced filter was highly porous structure, superior filtration properties with high removal efficiencies under low pressure [16]. MIL-125-NH_2_ was used for simultaneously monitor of methyl 1-naphthalene acetate, parathion-methyl, fenitrothion, bromophos and phenthoate residues in pomelo [17].

MIL-125-NH_2_ was added to catalase (CAT), and the immobilizing process depends on the adsorption and covalent binding between catalase and amino group of MIL-125-NH_2_. The obtained composite from catalase and MOFs achieved high recovery activity, pH stability and thermostability [18].

MIL-125-NH_2_ was added to silver molybdate (Ag_2_MoO_4_) and silver vanadate (AgVO_3_). The composite was tested as antimicrobial and anticancer agents. The obtained Ag_2_MoO_4_@MIL-125-NH_2_ achieved as antibacterial; the inhibition zone of Ag_2_MoO_4_@MIL-125-NH_2_ was higher than MIL-125-NH_2_ with seven times high. Moreover, the anticancer activity of Ag_2_MoO_4_@MIL-125-NH_2_ displaied 1.58 times more than MIL-125-NH_2_ [19].

MIL-125-NH_2_ was connected with nerve agent antidote (2-[(hydroxyimino)methyl]-1-methyl-pyridinium chloride) via simple, safe and low-cost synthetic approach. The mechanism of encapsulation into the pores of MIL-125-NH_2_ was related to the interactions between the nerve agent antidote and the pore walls via π-π stacking and hydrogen bonds [20]. Polysaccharides are a regulators for nanoparticles. Moreover, polysaccharides are a biocompatible, biodegradable and noncorrosive biopolymers. Carboxymethyl cellulose (CMC) is the excellent example of functionalized and edible cellulose derivative that is congaing side branch carboxylate group [4, 21, 22]. Additionally, CMC is characteristics with a unique features that are pushed up in many food industries and pharmaceutical industries such as edibility, biodegradability, non-toxic, water soluble, thinking agent and other compatibility with human cells and no allergy as well [23–26]. Indeed, the nanoparticles that have a bioactive properties usually need a formulation in which the activity of nanoparticles is amplified. Obviously, in this current work, a bioactive MOF (MIL-125-NH_2_) was formulated in CMC and decorated with thiophene to be used as antimicrobial and antiviral as well as anticancer active agent with great biocompatibility.

## Experimental section

### Reagents

2-Aminoterephthalic acid (99%, Aldrich), titanium isopropoxide (Merck), *N,N*-dimethylformamide (99.9%, Aldrich), chloroform (99.5%, Aldrich), toluene (PA, Fisher Scientific), thiophene carboxaldehyde (99%, Aldrich).

### Synthesis of MIL-125-NH_2_

The current MOFs were prepared by adding 1 mL of titanium isopropoxide to a solution of 0.37 g 2-aminoterephthalic acid in *N,N*-dimethylformamide/methanol (9:1 v/v) (50 mL) at room temperature. The mixture was added in autoclave, closed and placed in the oven at 150 °C for 18 h. The yellow solids were separated, washed with DMF several times, CHCl_3 _several times, and then immersed into ethanol for three night replaced DMF guest molecules.

### Synthesis of MIL-125-NH_2_@CMC

Alcoholic solution (15 mL) of CMC (656 mg, 4 mmol) was added drop wisely at room temperature to MIL-125-NH_2_ (1.0 g, 3.3 mmol NH_2_ equivalents) dispersed in ethanol (15 mL), and the solution was stored for 5 days at room temperature. The obtained composites were then washed with ethanol several times.

### Synthesis of sulfur-containing composite

The composite (Thio@MIL-125-NH_2_**@**CMC) was prepared by adding alcoholic (15 mL) solution of thiophene carboxaldehyde (1 mmol) drop wisely at room temperature to MIL-125-NH_2_@CMC (1 mmol) in ethanol (15 mL), and the resulting solution was standed for 3 days at room temperature. The obtained composite was washed with ethanol and dried in open air.

### Characterizations

Physiochemical analysis was included; Fourier-transform infrared spectroscopy (FTIR) using Spectrum Two IR Spectrometer—PerkinElmer, Inc., Shelton, USA”. Spectral analysis was obtained at 32 scans and 4 cm^−1^ resolutions in wavenumbers ranging from 4000 to 400 cm^−1^. The powder X-ray diffraction (PXRD) was carried out using a Diano X-ray diffractometer (Philips) provided with a CuKα radiation source (*λ* = 0.15418 nm), energized at 45 kV, as well as a generator (PW 1930) and a goniometer (PW 1820). On the other side, the topography and particle size were carried out using Field emission scanning electron microscopy (FESEM), Quanta FEG 250, FEI, Republic of Czech), which was connected with energy-dispersive X-ray spectrometry (EDAX) and transmission electron microscopy (TEM) JEOL-JEM-1230. The dynamic light scattering was carried out to investigate the average diameter, the size distribution that measured by using a particle size analyzer (Nano-ZS, Malvern Instruments Ltd., UK).

### Antimicrobial activity

The antimicrobial activity of MIL-125-NH_2_@CMC and Thio@MIL-125-NH_2_@CMC against four microorganisms, including *Escherichia coli* ATCC25922, *Staphylococcus aureus* ATCC25922, *Candida albicans* ATCC90028 and *Aspergillus niger* RCMB 02,724, was evaluated using the agar well diffusion technique. Minor modifications were made to the M51-A2 document of the Clinical Laboratory Standard Institute [27] when performing the agar diffusion test. MIL-125-NH_2_@CMC and Thio@MIL-125-NH_2_@CMC, standard antibiotic (Amoxicillin/clavulanate), were added to agar wells (7 mm) at a concentrations of 1000 g/mL individually, followed by incubation at 37 °C for 24 h. On the other hand, fungal strains were cultivated on PDA plates and incubated at 30 °C for 3–5 days. The fungal suspension was prepared in sterilized phosphate buffer solution (PBS) pH 7.0, and after counting in a cell counter chamber, the inoculums were adjusted to 10^7^ spores/mL. One milliliter was distributed equitably across agar PDA Plates. Then, 100 µl of the tested MIL-125-NH_2_@CMC and Thio@MIL-125-NH_2_@CMC and reference antifungal (Nystatin) at concentration 1000 g/mL were added. After incubating all PDA plates at 30 °C for 72 h, the diameter of the inhibition zone was measured. To determine minimum inhibitory concentrations of MIL-125-NH_2_@CMC, and Thio@MIL-125-NH_2_@CMC. AMC and Nystatin were prepared in various concentrations ranging from 1000 to 3.9 g/mL and then tested separately against selected bacterial and fungal strains [28, 29].

### Antiviral activity

Two viruses Herpes simplex 1 (HSV1) and Coxsackie B4 (COX B4) were used in this experiment. Cells were seeded into 200 µL of medium in a 96-well plate and incubated overnight at 37 °C and 5% CO_2_ to enable the cells to adhere to the wells. Incubate virus suspension and maximum non-toxic concentration of tested compounds in equal volume (1:1 v/v) for 1 h, then centrifuge 100 µL of virus suspension at 150 rpm for 5 min and incubate for 24 h. After incubation, 20 µL was added to each well, shaken for 5 min at 150 rpm, and then incubated at 37 °C for 1 to 5 h to enable the MTT to be metabolized. The optical density was measured at 560 nm, and the background was subtracted at 620 nm [30].

### In vitro cytotoxicity and anticancer activity

The cytotoxicity of prepared MIL-125-NH_2_@CMC, and Thio@MIL-125-NH_2_@CMC against Vero (ATCC-CCL-81), MCF7 (breast cancer), and PC3 (prostate cancer) cell lines was assessed using the 3-(4,5-dimethylthiazol-2-yl)-2,5-diphenyltetrazolium bromide assay (MTT) [31, 32]. The following formula was used to calculate cell quantity and viable cell percentage [33].

$$\mathrm{Viability\, \%}= \frac{\mathrm{Test \,OD}}{\mathrm{Control \,OD}}\mathrm{ X }100$$

$$\mathrm{Inhibition\, \%}=100-\mathrm{Viability\, \%}$$

## Results and Discussion

**Scheme 1:**
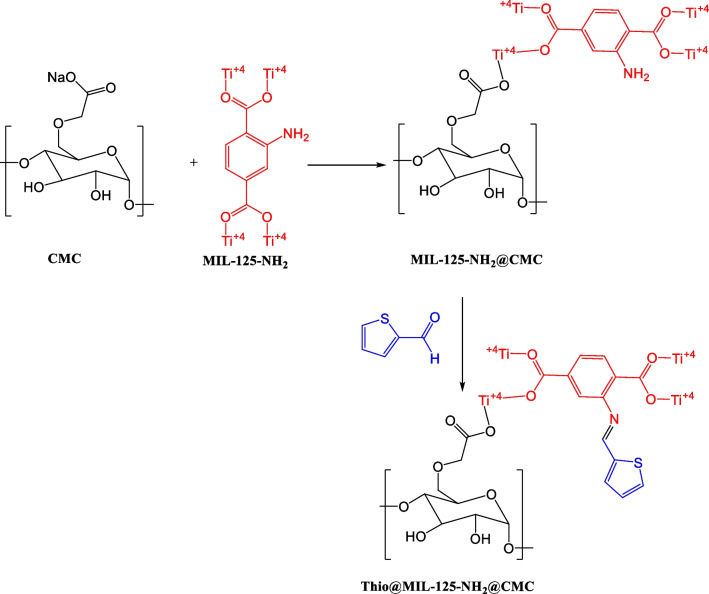
The reaction details of the MOF formulation using CMC and thiophene

The free amino group attached to the backbone of MIL-125-NH_2_ was used for the preparation of S-functionalized MOFs (Thio@MIL-125-NH_2_**@**CMC) as illustrated in Scheme 1. Post-synthetic modification (PSM) technique was used to synthesis of target composites, 2-thiophene carboxylaldehyde was added to MIL-125-NH_2_**@**CMC by PSM strategy. The novel synthesized Thio@MIL-125-NH_2_**@**CMC showed interesting properties such as high stability, large surface area and wide pore size. Thio@MIL-125-NH_2_**@**CMC having the imine (C=N) group obtained by covalent bonding between 2-thiophene carboxaldehyde and free amino group of MIL-125-NH_2_. This kind of imine (C=N) groups cannot be formed by direct preparation, but using PSM technique it can be formed. The obtained sulfur functionalized MOFs (S-MOFs) composite was extended to apply in drug delivery system such as antimicrobial and anticancer.

**Fig. 1:**
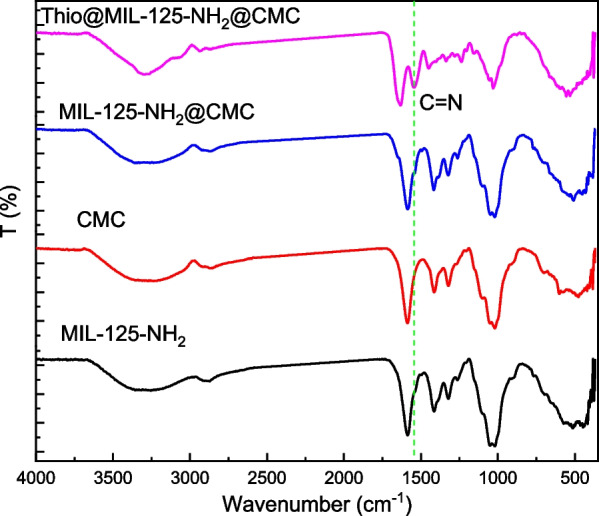
FTIR of prepared nanocomposite and their neat materials

The physiochemical characterizations of prepared samples were carried out using FTIR and PXRD crystallography. The FTIR spectra for formula and its neat materials are shown in Fig. 1. The MIL-125-NH_2_ spectrum was observed with a broad band at 3339 cm^−1^ that attributed to υ_asym_ (N–H) and bands at 1586 and 1415 cm^−1^ corresponding to the benzene rings function groups included C=C stretching modes of the aromatic ring and C–N stretching vibrations [34]. Additionally, bands at 1328 cm^−1^ are due to carboxylate groups and 1263 responding to C-N stretching vibrations[20]. Otherwise, the O−Ti−O bonds were appeared as small bands at 773 and 641 cm^−1^ [35]. On the other hand, the CMC spectrum was represented a specific characteristic bands at 3275, 2872, 1592, 1415 and 1022 cm^−1^ are determined to the stretching vibration of hydroxyl groups, CH, COO- asymmetric, symmetric and carbohydrate linkage, respectively [36, 37]. Whereas, the MIL-125-NH_2_@CMC spectrum observed obvious change in function groups values in comparison with its neat materials, the hydroxyl group band was shifted to lower frequency (3260 cm^−1^) as well as the bands at 1257 cm^−1^ was observed with a new position for C-N stretching vibrations. Otherwise, the Thio@MIL-125-NH_2_@CMC nanocomposite was shown the sharpest hydroxyl group band at 3315 cm^−1^ in comparison with the neat materials. Additionally, a new bands were observed at 1651 and 1540 are related to thiophene interaction. Moreover, the characteristic bands of thiophen were assigned at shifted position at 556 cm^−1^ that corresponding to C-S-C stretching [38]. These above observations, change in bands passion as well as appearing of new bands, were affirmed the interaction between the neat materials and the target composites MIL-125-NH_2_@CMC and Thio@MIL-125-NH_2_@CMC.

**Fig. 2:**
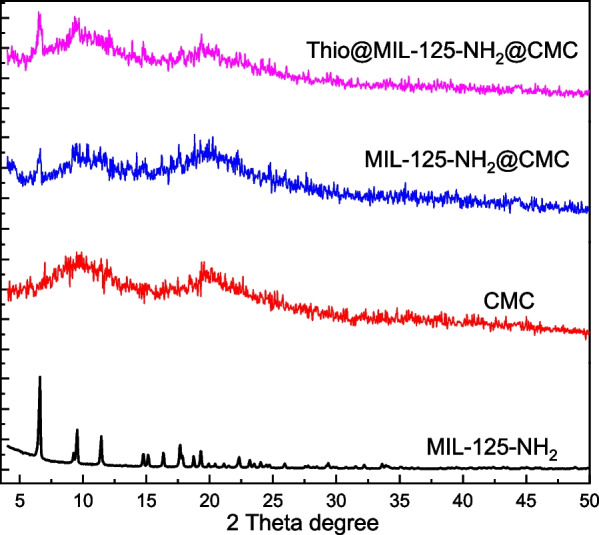
PXRD pattern prepared nanocomposite and their neat materials

Beside, the crystallographic pattern of prepared nanocomposite and their neat materials were shown in Fig. 2. MIL-125-NH_2_ showed diffraction pattern at 2θ = 6.5°, 9.5°, 11.4°, 16.2° and 17.6°, corresponding to (101), (200), (211), (222) and (312) indexation that is referred to formed phase of pure MIL-125-NH_2_ [39], and this was in agreement with typical MIL-125-NH_2_ PXRD pattern [40, 41]. However, the CMC pattern was observed two hubs peaks at around 10° and 19.5° that are referred to amorphous structure of CMC [42]. On the other side, the nanocomposite MIL-125-NH_2_@CMC pattern was obviously presented both peaks of MIL-125-NH_2_ and CMC. Instead, the CMC pattern was domain the spectrum while the MIL-125-NH_2_ clearly at 6.4° and 16.9°. Otherwise, the Thio@MIL-125-NH_2_@CMC pattern was affirmed a highest crystallinity in comparison with neat CMC and MIL-125-NH_2_@CMC. The Thio@MIL-125-NH_2_@CMC peaks were observed with high intensity at 6.5°and 11.9°confirming the presence of MIL-125-NH_2_ MOFs in the composite [43].

**Fig. 3:**
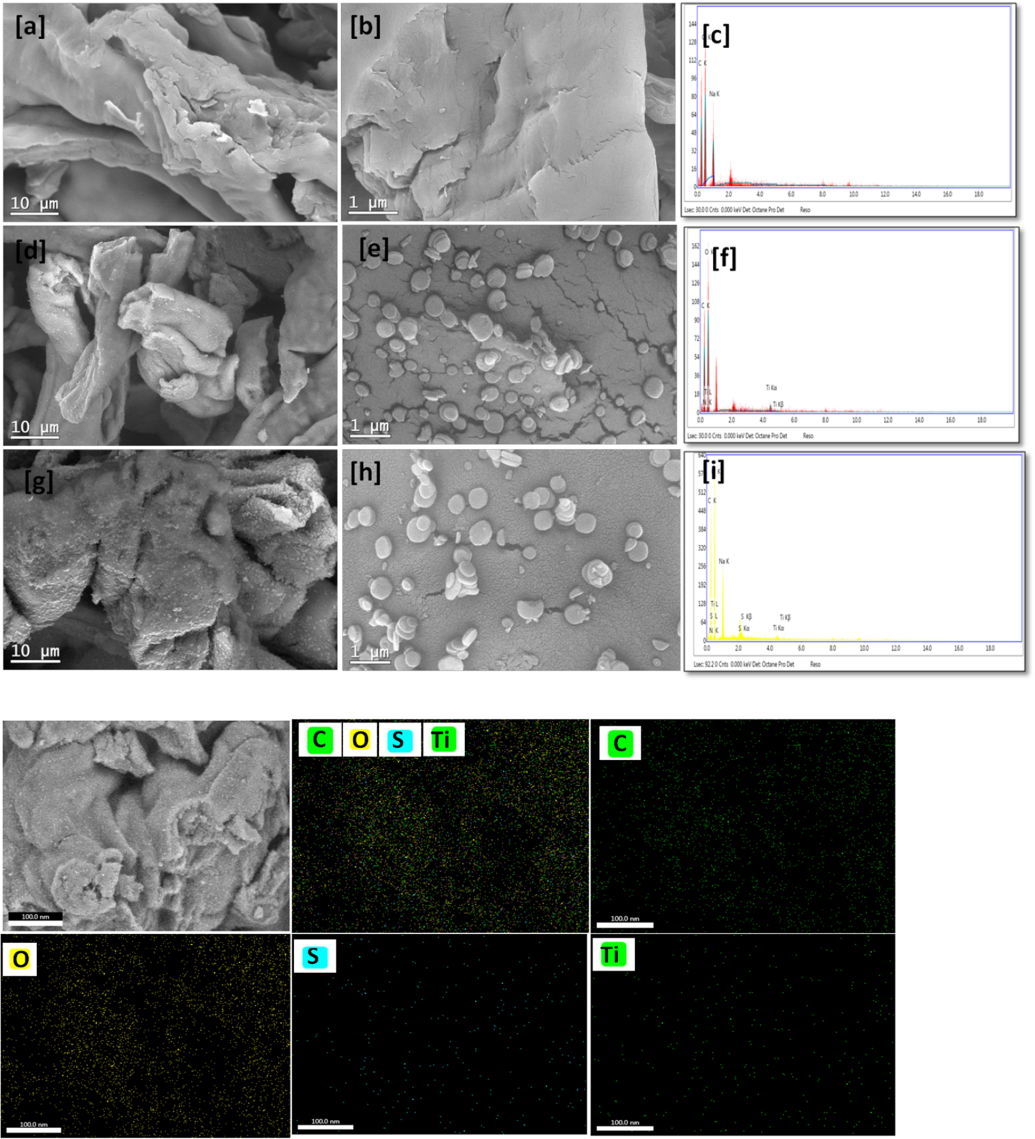
SEM images of CMC with low magnification (**a**), high magnification (**b**) and EDX chart (**c**). MIL-125-NH_2_@CMC SEM images with low magnification (**d**), high magnification (**e**) and EDX chart (**f**). Thio@MIL-125-NH_2_@CMC SEM images with low magnification (**g**), high magnification (**h**) and EDX chart (**i**) as well as mapping for (C, O, S and Ti ions) and separated chart for C, O,S and Ti

The topographical study was involved SEM, EDX and mapping as well as TEM and SEAD pattern. The SEM images are shown in Fig. 3 as low and high magnification with EDX chart for CMC, MIL-125-NH_2_@CMC and Thio@MIL-125-NH_2_@CMC provide with mapping. The CMC low magnification image **(**Fig. 3a**)** was observed as a typical CMC performance that mildly fibers and the high magnification image **(**Fig. 3b**)** clearly the fibers as a polymer-like behavior. In addition, the MIL-125-NH_2_@CMC image at low magnification **(**Fig. 3d**)** was observed as a CMC structure with protrusions that are obviously assigned in high magnification **(**Fig. 3e**)** as a particle that is referred to MIL-125-NH_2_. Moreover, the Thio@MIL-125-NH_2_@CMC nanocomposite surface morphology was observed with a unique surface morphology at low magnification (Fig. 3g**)** that was clear as a hairy surface with the protrusions as assigned previously in the sample MIL-125-NH_2_@CMC. However, this surface texture was disappeared in the high magnification image **(**Fig. 3h**)** but recorded as tiny parts over MIL-125-NH_2_ particles surface. In this context, the EDX chart of CMC **(**Fig. 3c**)** was shown a typical elemental content of CMC, namely carbon, oxygen and sodium. Besides, the EDX chart of MIL-125-NH_2_@CMC **(**Fig. 3f**)** affirmed the elements added to CMC including nitrogen and titanium that are due to MIL-125-NH_2_. However, the Thio@MIL-125-NH_2_@CMC EDX chart **(**Fig. 3i**)** appeared with all elements mentioned above with sulfur. Furthermore, the mapping charts clarified the distributions of titanium and sulfur ions that are observed with a homogeneous distribution over the Thio@MIL-125-NH_2_@CMC nanocomposites surface.

**Fig. 4:**
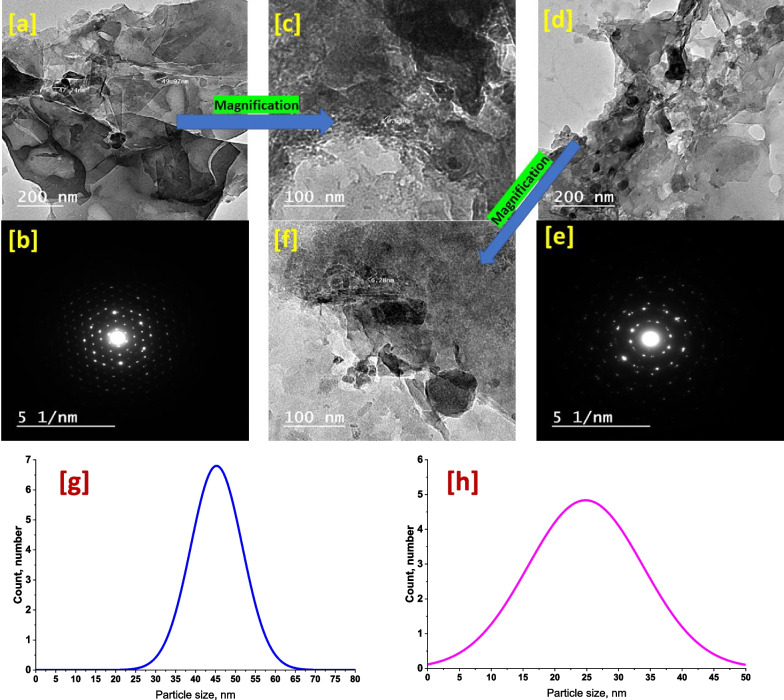
TEM image of MIL-125-NH_2_@CMC with low magnification (**a**) and high magnification (**c**) and SEAD pattern (**b**) as well as Thio@MIL-125-NH_2_@CMC with low magnification (**d**) and high magnification (**f**) and SEAD pattern (e). The particle size distribution of MIL-125-NH_2_@CMC (**g**) and Thio@MIL-125-NH_2_@CMC (h)

The TEM images as well as the SEAD pattern of MIL-125-NH_2_@CMC and Thio@MIL-125-NH_2_@CMC nanocomposites are shown in Fig. 4. The MIL-125-NH_2_@CMC at low magnification (Fig. 4a) observed a layer arranged as stacked on top of each other and doped with circulated particles which both in nanoscale. Indeed, the layers are referred to CMC and the particles are referred to MIL-125-NH_2_. Moreover, the high magnification image (Fig. 4c) observed the nanocomposite component interacted together and the average particle size was around 50 nm as well as the particle size distribution was affirmed that as shown in (Fig. 4g). Additionally, the SEAD pattern (Fig. 4b) affirmed the polycrystalline nature of nanocomposite. On the other hand, the Thio@MIL-125-NH_2_@CMC image at low magnification (Fig. 4d) revealed exceptional in this nanocomposite behavior where the particles interacted obviously with each other at low magnification image and the layers were expanded to involve the particle. Otherwise, at the high magnification image (Fig. 4f), the nanocomposite is clearly assigned as a single part arranged in different directions. These observations may be related to the thiophene role that collected the nanocomposite component and incorporated both into each other. Moreover, the particle size of Thio@MIL-125-NH_2_@CMC was measured around 35 nm. These results are in a nice agreement with the particle size distribution (Fig. 4h). The SEAD (Fig. 4e) affirmed the above phenomena that showed polycrystalline behavior with the highest chain spots that referred to the high combination.

### Antimicrobial activity

**Fig. 5:**
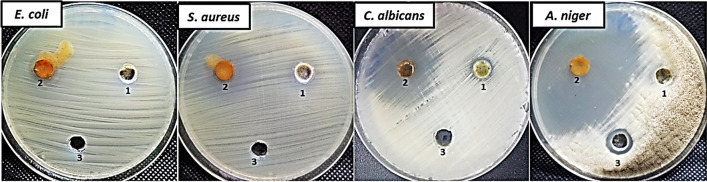
Antimicrobial activity of MIL-125-NH_2_@CMC **(1)** and Thio@MIL-125-NH_2_@CMC **(2)** and AMC/NS **(3)** using agar well diffusion method

Composites based on thiophene have received much attention in the last period due to the thiophene nucleus has been recognized as an important entity in the synthesis of heterocyclic compounds with promising pharmacological characteristics [44]. Therefore, in the current study, antimicrobial activity of MIL-125-NH_2_@CMC and Thio@MIL-125-NH_2_@CMC was evaluated against *E. coli, S. aureus, C. albicans* and *A. niger* as shown in Fig. 5 and Table 1. Results revealed that both MIL-125-NH_2_@CMC and Thio@MIL-125-NH_2_@CMC at concentration 1000 µg/ml exhibited weak antibacterial activity, where Thio@MIL-125-NH_2_@CMC was higher than MIL-125-NH_2_@CMC only. Also, inhibition zones of Thio@MIL-125-NH_2_@CMC were 10 and 17 mm against *E. coli* and *S. aureus*, respectively, while as AMC did not give any inhibition on both *E. coli* and *S. aureus*. Moreover, antibacterial results revealed that MICs of Thio@MIL-125-NH_2_@CMC were 1000 and 250 µg/ml against *E.coli* & *S. aureus*, respectively. On the other hand, Thio@MIL-125-NH_2_@CMC showed promising antifungal activity against both unicellular and multicellular fungi. Inhibition zones of Thio@MIL-125-NH_2_@CMC toward *C. albicans* and *A. niger* were 28 and 69 mm, respectively; but inhibition zones of MIL-125-NH_2_@CMC only were 12 and 10 mm, respectively. Furthermore, MICs of Thio@MIL-125-NH_2_@CMC toward *C. albicans* and *A. niger* were 31.25 and 0.97 µg/ml, respectively, but were 500 & 1000 µg/ml for MIL-125-NH_2_@CMC, respectively. This indicates adding of thiophene to MIL-125-NH_2_@CMC led to increase in antifungal activity. Previous studies reported that thiophene-based materials have antifungal activity against unicellular and multicellular fungi [45–47]. de Araújo Neto, de Lima, de Oliveira, de Souza, Buonafina, Anjos, Brayner, Alves, Neves and Mendonça-Junior [45] reported that 2-(5-nitro-thiophene)-thiosemicarbazones derivatives have antifungal activity against *Candida sp.* and *Cryptococcus neoformans* with inhibition of enzymes related to biosynthesis of ergosterol. Moreover, Wen, Jian, Shang and He [46] illustrated that thiophene-based stilbene derivatives exhibited antifungal activity against *Botrytis cinerea* where IC50 was in range 168.5 -155.4 µg/mL. Furthermore, Guimarães, Reis, Silva, Mendonça Junior, Converti, Pessoa Jr, Damasceno and Silva [47] revealed that microemulsion containing a thiophene derivative has potential antifungal activity against *Candida* species (MIC = 270–540 µg/mL) and *C. neoformans* (MIC = 17 µg/mL). Also, Mabkhot, Alatibi, El-Sayed, Al-Showiman, Kheder, Wadood, Rauf, Bawazeer and Hadda [48] reported that novel armed thiophene derivatives have antibacterial and antifungal activity against Gram-negative, Gram-positive bacteria, unicellular and multicellular fungi.

**Table 1:** Inhibition zones and minimum inhibitory concentrations of MIL-125-NH_2_@CMC and Thio@MIL-125-NH_2_@CMC toward *E. coli, S. aureus, C. albicans* and *A. niger*

Bacteria/fungal strain	MIL-125-NH_2_@CMC		Thio@MIL-125-NH_2_@CMC		AMC/NS	
	IZ*	MIC**	IZ	MIC	IZ	MIC
*E. coli*	ND	ND	10	1000	ND	ND
*S. aureus*	12	500	17	250	ND	ND
*C. albicans*	12	500	28	31.25	ND	ND
*A. niger*	10	1000	79	0.97	15	250

*Means inhibition zone (mm) at concentration 1000 µg/mL

**Means minimum inhibitory concentration (µg/mL)

### Antiviral activity

**Fig. 6:**
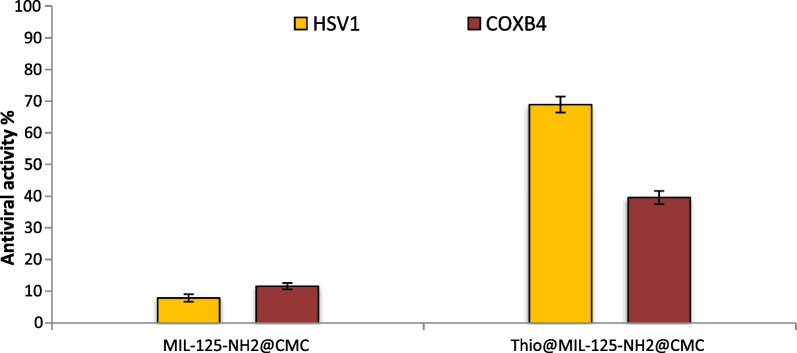
Antiviral activity of MIL-125-NH_2_@CMC and Thio@MIL-125-NH_2_@CMC against HSV1 and COX B4 viruses

Viral infections pose significant risks to public health, as evidenced by epidemics such as COVID-19 in humans and African Swine Fever (ASF) in animals, which justifies intensive research into the development of novel antiviral molecules and materials. Antiviral activity of MIL-125-NH_2_@CMC and Thio@MIL-125-NH_2_@CMC was evaluated against HSV1 and COX B4 human viruses as shown in Fig. 6. Antiviral activity of MIL-125-NH_2_@CMC and Thio@MIL-125-NH_2_@CMC was evaluated at maximum non-toxic concentration (MNTC), where MNTC of MIL-125-NH_2_@CMC and Thio@MIL-125-NH_2_@CMC against vero normal cell line was 250 and 125 µg/ml, respectively. Results revealed that Thio@MIL-125-NH_2_@CMC exhibited promising antiviral activity against both HSV1 and COX B4 where showed activity toward HSV1 higher than COX B4. Moreover, antiviral activity of Thio@MIL-125-NH_2_@CMC toward HSV1 was 68.89%, while was 39.60% in the case of COXB4. On the other hand, MIL-125-NH_2_@CMC without thiophene exhibited very weak antiviral activity against HSV1 and COX B4 where inhibition percentages were 7.84 and 11.57%, respectively. Herein, Thio@MIL-125-NH_2_@CMC has promising antiviral activity against HSV1 and COX B4 which can be used in biomedical application. Jaros, Król, Bażanów, Poradowski, Chrószcz, Nesterov, Kirillov and Smoleński [49] synthesized bioMOF based on silver, 1,3,5-Triaza-7-Phoshaadamantane and Pyromellitic Acid, where it exhibited significant antiviral activity against human adenovirus 36 (HAdV-36). Furthermore, a new complex MIL-101(Fe)-T705 was created by combining the MOF material MIL-101(Fe) with the medication favipiravir (T-705), and this complex demonstrated good antiviral activity against Influenza Virus at low concentrations [50].

### Cytotoxicity and anticancer activity

**Fig. 7:**
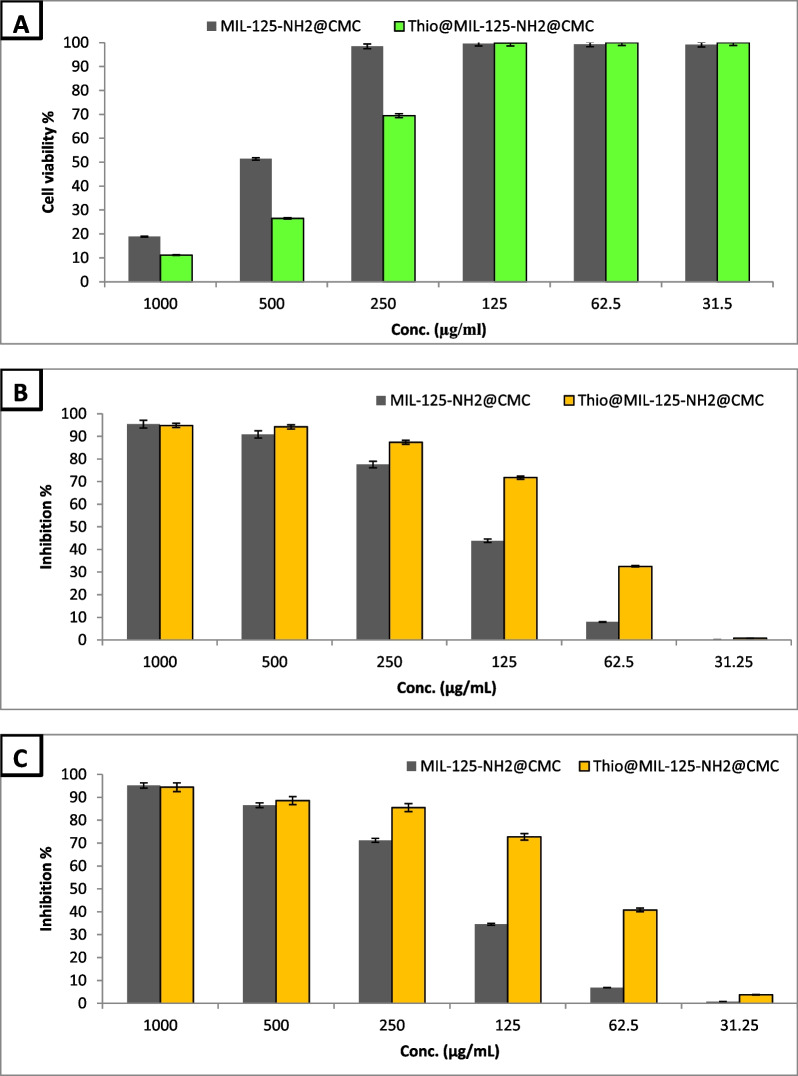
Cytotoxicity of MIL-125-NH_2_@CMC and Thio@MIL-125-NH_2_@CMC against Vero normal cell line (**A**), MCF7 (**B**) and PC3 (**C**) cancerous cell lines

In vitro cytotoxicity of compounds on human normal cell lines is considered the first step to detect the safety of these compounds [32]. In the current study, cytotoxicity of MIL-125-NH_2_@CMC and Thio@MIL-125-NH_2_@CMC on Vero normal cell line was evaluated as shown in Fig. 7A. Results illustrated that both MIL-125-NH_2_@CMC and Thio@MIL-125-NH_2_@CMC at concentrations up to 125 µg/mL did not show any toxicity on Vero normal cell line, also IC50 were 515 & 395 µg/mL, respectively. Generally, if the IC50 is ≥ 90 μg/mL, the material is classified as non-cytotoxic [51]. Therefore, both prepared MIL-125-NH_2_@CMC and Thio@MIL-125-NH_2_@CMC are safe in use for biological applications.

Recently, metal–organic frameworks (MOFs) have attracted attention as anticancer drug carriers, because of their low toxicity and high efficiency [52]. MOFs have key roles in cancer therapy, where they can be combined with photothermal therapy and photodynamic therapy to improve cancer treatment. MOFs are also specific carriers for medicine delivery. Furthermore, MOFs, when combined with RNA interference, can induce effective gene expression and can be combined with other anticancer techniques to increase therapeutic efficacy [53]. In the current study, anticancer activity of MIL-125-NH_2_@CMC and Thio@MIL-125-NH_2_@CMC against MCF7 and PC3 cancerous cell lines was evaluated as illustrated in Figs. 7 B and C. Results revealed that both MIL-125-NH_2_@CMC and Thio@MIL-125-NH_2_@CMC exhibited promising anticancer activity, where Thio@MIL-125-NH_2_@CMC was higher than MIL-125-NH_2_@CMC. Figure 7B illustrates Thio@MIL-125-NH_2_@CMC has potential anticancer activity above 50% at concentrations >  = 125 µg/ml against MCF7 cancerous cell line. Also, IC_50_ of MIL-125-NH_2_@CMC and Thio@MIL-125-NH_2_@CMC were 165.75 and 93.16 µg/ml against MCF7, respectively. Likewise, Thio@MIL-125-NH_2_@CMC exhibited promising anticancer activity toward PC3 cancerous cell line, where IC_50_ was 88.45 µg/ml. Also, MIL-125-NH_2_@CMC exhibited moderate anticancer activity against PC3 cancerous cell line, where IC_50_ was 182.78 µg/ml. Previous studies reported that sulfur-containing compounds have promising anticancer activity [54, 55]. Song, Huang, Mao, Chen, Wang, Yang, Liu, Zhang, Qiu and Chen [56] prepared MIL-125-TI-HA @ DOX, which demonstrated anticancer activity with no toxicity, where electrostatic interaction between DOX (Doxorubicin) amino groups and HA carboxyl groups, as well as the dual protective properties of MIL-125-Ti and HA on medicines, supported the increase in drug loading content.

## Conclusion

The biopolymer and MOF-based nanocomposites were prepared. The prepared nanocomposites, namely MIL-125-NH_2_@CMC and Thio@MIL-125-NH_2_@CMC, were characterized via physiochemical and topographical analysis. These analyses emphasized the prepared nanocomposite in nanoscale. The prepared composite (Thio@MIL-125-NH_2_@CMC) exhibited promising antifungal activity against unicellular and multicellular fungi. Likewise, it showed antibacterial activity against Gram-negative and Gram-positive bacteria. Moreover, it appeared promising antiviral activity against both HSV1 and COX B4 viruses at MNTC. Furthermore, it showed potential anticancer activity against MCF7 and PC3 cancerous cell lines, where IC_50_ were 93.16 and 88.45%, respectively.

## Data Availability

The data made available upon requested.

## References

[1] Prestinaci F, Pezzotti P, Pantosti A (2015). Antimicrobial resistance: a global multifaceted phenomenon. Pathog Global Health.

[2] Joseph TM, Kar Mahapatra D, Esmaeili A, Piszczyk Ł, Hasanin MS, Kattali M, Haponiuk J, Thomas S (2023). Nanoparticles: taking a unique position in medicine. Nanomaterials.

[3] Smith RD, Coast J (2002). Antimicrobial resistance: a global response. Bull World Health Organ.

[4] Hasanin MS. Cellulose‐based biomaterials: chemistry and biomedical applications, Starch‐Stärke, 2022:2200060

[5] Lashin I, Hasanin M, Hassan SA, Hashem AH. Green biosynthesis of zinc and selenium oxide nanoparticles using callus extract of Ziziphus spina-christi: characterization, antimicrobial, and antioxidant activity, Biomass Conv Biorefinery, 2021:1–14.

[6] Shehabeldine A, El-Hamshary H, Hasanin M, El-Faham A, Al-Sahly M (2021). Enhancing the antifungal activity of griseofulvin by incorporation a green biopolymer-based nanocomposite. Polymers.

[7] Cremonini F, Di Caro S, Covino M, Armuzzi A, Gabrielli M, Santarelli L, Nista EC, Cammarota G, Gasbarrini G, Gasbarrini A (2002). Effect of different probiotic preparations on anti-Helicobacter pylori therapy-related side effects: a parallel group, triple blind, placebo-controlled study. Am J Gastroenterol.

[8] Rosenberg SA, Yannelli JR, Yang JC, Topalian SL, Schwartzentruber DJ, Weber JS, Parkinson DR, Seipp CA, Einhorn JH, White DE (1994). Treatment of patients with metastatic melanoma with autologous tumor-infiltrating lymphocytes and interleukin 2. JNCI J Natl Cancer Inst.

[9] Anighoro A, Bajorath J, Rastelli GJJOMC (2014). Polypharmacology: challenges and opportunities in drug discovery: miniperspective. J Med Chem.

[10] Donawade DS, Raghu A, Muddapur U, Gadaginamath GS (2005). Chemoselective reaction of benz (g) indole based bisheterocycle dicarboxylate towards hydrazine hydrate: Synthesis and antimicrobial activity of new triheterocycles-5-pyrrolylaminocarbonyl/mercaptooxadiazolyl/4-allyl-5-mercaptotriazolylmethoxy-1-furfuryl-2-methylbenz (g) indoles. ChemInform.

[11] Donawade DS, Raghu A, Gadaginamath GS (2006). Synthesis and antimicrobial activity of some new 1-substituted-3-pyrrolyl aminocarbonyl/oxadiazolyl/triazolyl/5-methoxy-2-methylindoles and benz [g] indoles. ChemInform.

[12] Donawade DS, Raghu A, Gadaginamath GS. Synthesis and antimicrobial activity of novel linearly fused 5-substituted-7-acetyl-2, 6-dimethyloxazolo [4, 5-f] indoles, 2007.

[13] Nagaraja A, Jalageri MD, Puttaiahgowda YM, Raghava Reddy K, Raghu AV (2019). A review on various maleic anhydride antimicrobial polymers. J Microbiol Methods.

[14] Zhu Z, Bao L, Pestov D, Xu P, Wang W-N (2023). Cellular-level insight into biointerface: from surface charge modulation to boosted photocatalytic oxidative disinfection. Chem Eng J.

[15] Lin G, Zhang Y, Hua Y, Zhang C, Jia C, Ju D, Yu C, Li P, Liu J. Bioinspired metalation of the metal‐organic framework MIL‐125‐NH2 for NADH regeneration and gas‐liquid‐solid three‐phase enzymatic CO_2_ REDUCTION, Angewandte Chemie International Edition, 2022. 10.1002/anie.202206283 35585038

[16] Hao D, Fu B, Zhou J, Liu J (2022). Efficient particulate matter removal by metal-organic frameworks encapsulated in cellulose/chitosan foams. Sep Purif Technol.

[17] Zhang Q, Xiao W, Wu Y, Fan Y, Zou W, Xu K, Yuan Y, Mao X, Wang Y (2022). A simple, environmental-friendly and reliable d-SPE method using amino-containing metal–organic framework MIL-125-NH2 to determine pesticide residues in pomelo samples from different localities. Food Chem.

[18] Wang Z, Liu Y, Li J, Meng G, Zhu D, Cui J, Jia S (2022). Efficient immobilization of enzymes on amino functionalized MIL-125-NH2 metal organic framework. Biotechnol Bioprocess Eng.

[19] Abdelhameed RM, Abu-Elghait M, El-Shahat M (2022). Engineering titanium-organic framework decorated silver molybdate and silver vanadate as antimicrobial, anticancer agents, and photo-induced hydroxylation reactions. J Photochem Photobiol A.

[20] Vilela SM, Salcedo-Abraira P, Colinet I, Salles F, De Koning MC, Joosen MJ, Serre C, Horcajada P (2017). Nanometric MIL-125-NH2 metal–organic framework as a potential nerve agent antidote carrier. Nanomaterials.

[21] Diwakar B, Rajeswari D, Singh J, Haritha P, Srinivasa Rao S, Swaminadham V, Rao BT, Reddy V. Carboxymethyl cellouse stabilized cobalt sulfide nanoparticles: preparation, characterization and application, J Clust Sci, 2022:1–11.

[22] Kumar J, Singh J, Reddy V. Carboxymethyl cellulose stabilized lead sulfide nanocrystals: synthesis, characterization and catalytic applications, 2021.

[23] Upadhyaya L, Singh J, Agarwal V, Pandey A, Verma SP, Das P, Tewari R (2014). In situ grafted nanostructured ZnO/carboxymethyl cellulose nanocomposites for efficient delivery of curcumin to cancer. J Polym Res.

[24] Upadhyaya L, Singh J, Agarwal V, Pandey A, Verma SP, Das P, Tewari R (2015). Efficient water soluble nanostructured ZnO grafted O-carboxymethyl chitosan/curcumin-nanocomposite for cancer therapy. Process Biochem.

[25] Upadhyaya L, Singh J, Agarwal V, Tewari RP (2013). Biomedical applications of carboxymethyl chitosans. Carbohyd Polym.

[26] Upadhyaya L, Singh J, Agarwal V, Tewari RP (2014). The implications of recent advances in carboxymethyl chitosan based targeted drug delivery and tissue engineering applications. J Control Release.

[27] NCFCL Standards, Reference method for broth dilution antifungal susceptibility testing of yeasts, National Committee for Clinical Laboratory Standards Wayne, PA, 2002.

[28] Valgas C, Souza SMD, Smânia E, Smânia A (2007). Screening methods to determine antibacterial activity of natural products. Braz J Microbiol.

[29] Hashem AH, Khalil AMA, Reyad AM, Salem SS (2021). Biomedical applications of mycosynthesized selenium nanoparticles using *Penicillium*
*expansum* ATTC 36200. Biol Trace Elem Res.

[30] Desselberger U. Antiviral Methods and Protocols. Methods in Molecular Medicine, Volume 24: Kinchington D, Schinazi RF, eds. ($99.00.) Humana Press, 2000. ISBN 0 896 03561 1, Mol Pathol 53(3) (2000) 163–163.

[31] Van de Loosdrecht A, Beelen R, Ossenkoppele G, Broekhoven M, Langenhuijsen M (1994). A tetrazolium-based colorimetric MTT assay to quantitate human monocyte mediated cytotoxicity against leukemic cells from cell lines and patients with acute myeloid leukemia. J Immunol Methods.

[32] Khalil A, Abdelaziz A, Khaleil M, Hashem A (2021). Fungal endophytes from leaves of Avicennia marina growing in semi-arid environment as a promising source for bioactive compounds. Lett Appl Microbiol.

[33] Akl EM, Dacrory S, Abdel-Aziz MS, Kamel S, Fahim AM. Preparation and characterization of novel antibacterial blended films based on modified carboxymethyl cellulose/phenolic compounds, Polym Bull under press, 2020.

[34] Rodríguez NA, Savateev A, Grela MA, Dontsova D (2017). Facile synthesis of potassium poly (heptazine imide)(PHIK)/Ti-based metal–organic framework (MIL-125-NH2) composites for photocatalytic applications. ACS Appl Mater Interfaces.

[35] Choe J, Yang X, Yu J, Jang K, Kim M, An K (2022). Visible-light responsive PPynt@ NH2-MIL-125 nanocomposite for efficient reduction of Cr (VI). Colloids Surf A.

[36] Hebeish A, Hashem M, Abd El-Hady M, Sharaf S (2013). Development of CMC hydrogels loaded with silver nano-particles for medical applications. Carbohydr Polym.

[37] Turky G, Moussa MA, Hasanin M, El-Sayed NS, Kamel S. Carboxymethyl cellulose-based hydrogel: dielectric study, antimicrobial activity and biocompatibility, Arab J Sci Eng. 2020, 1–14.

[38] Sahin E, Camurlu P, Toppare L, Mercore VM, Cianga I, Yagˇcı Y (2005). Conducting copolymers of thiophene functionalized polystyrenes with thiophene. J Electroanal Chem.

[39] Wang P, Lang J, Liu D, Yan X (2015). TiO 2 embedded in carbon submicron-tablets: synthesis from a metal–organic framework precursor and application as a superior anode in lithium-ion batteries. Chem Commun.

[40] Qiu J, Yang L, Li M, Yao J (2019). Metal nanoparticles decorated MIL-125-NH2 and MIL-125 for efficient photocatalysis. Mater Res Bull.

[41] Sánchez NC, Guzmán-Mar JL, Hinojosa-Reyes L, Palomino GT, Cabello CP (2019). Carbon composite membrane derived from MIL-125-NH2 MOF for the enhanced extraction of emerging pollutants. Chemosphere.

[42] Ezati P, Rhim J-W, Moradi M, Tajik H, Molaei R (2020). CMC and CNF-based alizarin incorporated reversible pH-responsive color indicator films. Carbohyd Polym.

[43] Yamamoto T, Kokubo H, Kobashi M, Sakai Y (2004). Alignment and field-effect transistor behavior of an alternative π-conjugated copolymer of thiophene and 4-alkylthiazole. Chem Mater.

[44] Mabkhot YN, Kaal NA, Alterary S, Al-Showiman SS, Farghaly TA, Mubarak MS (2017). Antimicrobial activity of thiophene derivatives derived from ethyl (E)-5-(3-(dimethylamino) acryloyl)-4-methyl-2-(phenylamino) thiophene-3-carboxylate. Chem Cent J.

[45] de Araújo Neto LN, de Lima MDCA, de Oliveira JF, de Souza ER, Buonafina MDS, Anjos MNV, Brayner FA, Alves LC, Neves RP, Mendonça-Junior FJB (2017). Synthesis, cytotoxicity and antifungal activity of 5-nitro-thiophene-thiosemicarbazones derivatives. Chemico-Biol Interact.

[46] Wen L, Jian W, Shang J, He D (2019). Synthesis and antifungal activities of novel thiophene-based stilbene derivatives bearing an 1, 3, 4-oxadiazole unit. Pest Manag Sci.

[47] Guimarães GP, Reis MYDFA, Silva DTCD, Mendonça Junior FJB, Converti A, Pessoa A, Damasceno BPGDL, Silva JAD (2014). Antifungal activity of topical microemulsion containing a thiophene derivative. Braz J Microbiol.

[48] Mabkhot YN, Alatibi F, El-Sayed NNE, Al-Showiman S, Kheder NA, Wadood A, Rauf A, Bawazeer S, Hadda TB (2016). Antimicrobial activity of some novel armed thiophene derivatives and petra/osiris/molinspiration (POM) analyses. Molecules.

[49] Jaros SW, Król J, Bażanów B, Poradowski D, Chrószcz A, Nesterov DS, Kirillov AM, Smoleński P (2020). Antiviral, antibacterial, antifungal, and cytotoxic silver (I) BioMOF assembled from 1, 3, 5-triaza-7-phoshaadamantane and pyromellitic acid. Molecules.

[50] Xu M, Li X, Zheng H, Chen J, Ye X, Liu T (2022). Anti-influenza virus study of composite material with MIL-101 (Fe)-adsorbed favipiravir. Molecules.

[51] Ioset, J-R, Brun R, Wenzler T, Kaiser M, Yardley V. Drug screening for kinetoplastids diseases, a training manual for screening in neglected diseases. 2009.

[52] Batool M, Tawakkul N, Batool S, Zafar MN, Nazar MF (2023). Porous metal–organic framework nanoscale carriers as a potential platform for drug delivery.

[53] Zheng Y, Zhang X, Su Z (2021). Design of metal–organic framework composites in anti-cancer therapies. Nanoscale.

[54] E. De Gianni, C. Fimognari, Anticancer mechanism of sulfur-containing compounds, The Enzymes, Elsevier2015, pp. 167-192. 10.1016/bs.enz.2015.05.003 26298460

[55] Cerella C, Dicato M, Jacob C, Diederich M (2011). Chemical properties and mechanisms determining the anti-cancer action of garlic-derived organic sulfur compounds. Anti-Cancer Agents Med Chem Formerly Curr Med Chem-Anti-Cancer Agents..

[56] Song J-L, Huang Z-Q, Mao J, Chen W-J, Wang B, Yang F-W, Liu S-H, Zhang H-J, Qiu L-P, Chen J-H (2020). A facile synthesis of uniform hollow MIL-125 titanium-based nanoplatform for endosomal esacpe and intracellular drug delivery. Chem Eng J.

